# Assessing youth-friendly sexual and reproductive health services: a systematic review

**DOI:** 10.1186/s12913-018-2982-4

**Published:** 2018-03-27

**Authors:** Amanda Mazur, Claire D. Brindis, Martha J. Decker

**Affiliations:** 1 Philip R. Lee Institute for Health Policy Studies and Bixby Center for Global Reproductive Health, University of California, San Francisco, 3333 California Street, Suite 265, San Francisco, CA 94143-0936 USA; 2 Adolescent and Young Adult Health National Resource Center, University of California, San Francisco, 3333 California Street, Suite 245, San Francisco, CA 94143-0503 USA

**Keywords:** Youth-friendly, Adolescent, Sexual and reproductive health services, Measurement, Indicators, Global

## Abstract

**Background:**

Over the last quarter century, there has been an emergence of evidence-based research directed toward the development, implementation, and assessment of youth-friendly health services (YFHS) to improve the delivery of sexual and reproductive health services for young people. Despite these research efforts, evidence supporting the effectiveness of YFHS is limited, which may be attributed to a lack of consensus on how to define and measure youth-friendliness to track progress and evaluate outcomes. The purpose of this systematic review is to assess how youth-friendly sexual and reproductive health services are measured worldwide.

**Methods:**

We conducted a systematic review of studies measuring youth-friendly sexual and reproductive health services at health facilities published between January 2000 and June 2015 using PubMed, Web of Science, and POPLINE databases. Additional studies were identified by reviewing references of selected articles. Studies were screened to identify measurements and indicators that have been used to measure YFHS.

**Results:**

Our review identified 20 studies from an initial search of more than 11,000 records, including six from high-income countries and 14 from low-and middle-income countries. The review identified 115 indicators used for measuring youth-friendly sexual and reproductive health services. Our review found a lack of consistency in the tools and indicators used to measure YFHS. The three most frequently assessed domains were accessibility, staff characteristics and competency, and confidentiality and privacy. The majority of the indicators were not specific to young people’s needs and often reflected basic standards of care.

**Conclusions:**

This review shows the need for standardization and prioritization of indicators for the evaluation of YFHS. The results can be used to identify a core set of indicators that can be incorporated into a framework for assessing youth-friendly sexual and reproductive health services. There is a need to further distinguish between those variables that may have greatest impact on the use of services by young people, such as respect and privacy, those that impact the quality of services offered, and those that have limited relevance. Conducting more rigorous studies using a refined set of indicators is critical to measure and compare the impact and effectiveness of YFHS efforts.

## Background

Although there has been momentum in implementing sexual and reproductive health services (SRH) services in most countries, young people typically remain underserved by these services despite their demonstrated need [1, 2]. In a study of 70 low and middle income countries (LMICs), almost all the countries reported that only 10% or fewer of all adolescent women had visited a health facility in the past 12 months and were informed about family planning [2]. Moreover, 20 to 25% of married adolescents reported an unmet need for contraception according to data from 41 countries [3]. Although adolescents are at an increased risk for STIs and HIV infection in comparison to any other age group [4–6], adolescents face major barriers in accessing HIV testing and treatment. In sub-Saharan Africa, only 10% of young men and 15% of young women were aware of their HIV status [7]. Even when young people are able to access services, they may feel embarrassed, face stigma on sexual matters, or have concerns about judgmental providers [1, 8]. Youth-friendly health services (YFHS) are a promising approach to delivering health services to meet the SRH needs of young people [3].

Young people require services that support their physiological, cognitive, emotional, and social transition into adulthood [9, 10]. Delivering quality services that are tailored to young people may improve service use, adherence to contraceptive methods, and increase the likelihood of obtaining ongoing care [11, 12]. Therefore, understanding how to best deliver services to young people and evaluating the impact of service delivery is essential to improving youth SRH outcomes. According to the World Health Organization’s (WHO) 2001 Global Consultation on Adolescent Friendly Health Services, SRH services for adolescents should aim to achieve at least one of three goals: (1) provide a supportive environment, (2) improve reproductive health knowledge, attitudes, skills and behaviors, and (3) increase utilization of health and related services [13]. The WHO guidelines for providing YFHS recommends services that are accessible, acceptable, equitable, appropriate and effective [14].

Despite these general guidelines, there is a lack of consensus on what aspects of YFHS are most relevant and important to meet the health needs of young people [15, 16]. Furthermore, several systematic reviews of youth-friendly interventions found insufficient evidence to support the effectiveness of youth-friendly health interventions [15, 17].

Understanding how YFHS are defined and measured may clarify not only how to deliver appropriate services, but also how to assess if these services are effective and to compare different YFHS programs. Although a previous systematic review has assessed the measurement of youth-friendly services at the primary and tertiary levels from a youth-only perspective [18], no studies to our knowledge have focused specifically on the measurement of SRH services for young people, which may have specific needs [18], such as stigma and embarrassment, associated with sexual activity in this age group [19]. This study expands on previous literature to focus on how youth-friendly SRH services are measured worldwide and to identify commonly used indicators from the selected studies that potentially could be used to help develop a standardized method for in the assessing youth-friendly SRH services.

## Methods

We conducted a systematic review of peer-reviewed published studies measuring youth-friendly SRH services. Because the purpose of this review was to identify indicators that have been used to assess youth-friendliness and not to synthesize the findings on the impact, we included both qualitative and quantitative studies. We included qualitative methods as well as quantitative as this type of methodology can be used to assess constructs that cannot always be quantified and that may identify context-specific issues [20]. We searched for studies published in English between January 2000 and June 2015 on PubMed, Web of Science Core, and POPLINE. Other sources were identified through a snowball method of scanning references in selected sources. The search was conducted using search terms related to youth-friendly health services. Search terms used were “adolescent”, “youth”, “teen”, “teenagers”, “young”, “health services”, “friendly”, “health access”, “clinics”, “health delivery”, and “health center”.

We included studies related to the youth-friendliness of SRH services serving clients aged 10 to 24 years. In cases where studies included individuals aged 24 to 29 years, inclusion criteria was met as long as young people aged 10 to 24 years were the primary population focused on in the study and individuals aged older than 24 years were using facilities for continuation of care or partner specific care. Studies of young people receiving primary health care services were accepted based on the assumption that youth-friendly SRH services are components of primary health care. Abstracts were reviewed for inclusion of SRH services such as HIV, pregnancy, contraception, and sexually transmitted infections (STIs). Excluded studies were specifically related to specialty care such as mental health or tertiary care. If a study was not related to young people receiving care at an existing health facility, such as care received through outreach and education activities, the study was excluded. Studies conducting needs assessments for potential services also were excluded.

Two researchers screened each title and abstract and selected relevant studies based on inclusion and exclusion criteria. If a discrepancy arose, the two researchers reviewed the full article and came to a consensus noting reasons for inclusion or exclusion of the article. During the full review, if articles were missing data, such as detailed methods, questionnaires, or descriptions of measurements, such as surveys or interview guides, the corresponding authors were contacted by email to obtain missing information. No risk of bias or quality assessments were used because this review aims to describe the literature on how YFHS are measured and not to determine the size of an effect or compare study results.

We abstracted data into the data collection table from articles selected for the systematic review to summarize the characteristics of the articles based on setting, study design, instrument used, participants, and indicators used in the studies. As part of the systematic review, indicators used to measure youth-friendliness in selected articles were gathered and synthesized into corresponding domains. The initial domains used in this study were adapted from the WHO *Quality Assessment Guidebook: A Guide To Assessing Health Services For Adolescents* and Pathfinder’s *Clinic Assessment Guidebook*: A *Tool For Assessing And Improving Sexual And Reproductive Health Services for Youth* [21, 22]. The domains used from the WHO tool are “accessible”, “acceptable”, “appropriate”, “equitable”, and “effective” [21]. From the Pathfinder tool, we used “youth involvement”, “services provided”, “environment”, and “educational activities” [22]. Two additional domains, “staff characteristics and competency” and “confidentiality and privacy,” were added by the researchers as a number of indicators were more appropriately grouped under these new domains and to incorporate indicators that did not fit into the predefined domains. Both researchers reviewed placement of indicators in the corresponding domains and if a discrepancy arose, the researchers reached an agreement through additional discussion.

## Results

**Fig. 1:**
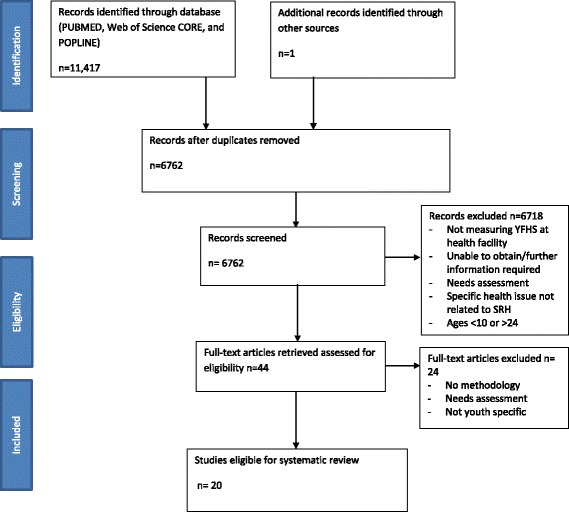
PRISMA Flowchart for article review process

Our initial search (Fig. 1) yielded 6762 unduplicated records. All titles and abstracts were initially screened by two researchers. Reasons for exclusion at this stage included not specifically measuring youth-friendliness, needs assessments for not yet existing facilities, not related to young people’s SRH, or the sample population was not within the age range of our inclusion criteria. We then retrieved 44 articles for full text review. The full review resulted in the final selection of 20 studies that met all of the inclusion criteria (Table 1). Studies included five mixed method studies [23–27], five qualitative studies [28–32], two case-control studies [33, 34], three randomized controlled trials (RCTs) [35, 36], and six other quantitative studies [12, 37–41]. All studies that used RCTs or case-control designs were located in LMICs.

**Table 1:** Summary of Studies Included in the Review

Author	Setting^a^	Study design	Participants	Domains^b^	Outcomes measured
Alli F. et al. (2013) [28]	South Africa; Upper middle income; Youth friendly university clinic	Cross-sectional study 1. In-depth interviews with managers and senior staff 2. Exit interviews with youth	200 youth aged 18–24 years 4 in-depth interviews with clinic managers and senior staff	1,2,5–8	Perception
Baumgartner et al. (2012) [37]	Kenya; Low income; HIV voluntary testing and counselling (VCT) youth friendly and non-youth friendly clinics	Repeated cross-sectional study 1. Baseline and 3 month follow-up client interviews 2. Baseline provider interviews structured observations of facilities	277 youth aged 15–24 years 46 providers 20 clinic observations	1–4, 6–8,12	Contraceptive use
		Adapted from Brindis et al. (2005) [23], Mmari and Magnani (2003) [33], and UNAIDS 2000 indices for youth-friendliness			
Brindis et al. (2005) [23]	USA; High income; 10 Primary care facilities, SRH clinics and alternative settings	Pre-post evaluation study 1. Semi-structured interviews with administrator/provider 2. Questionnaire for administrators 3. Exit survey questionnaires for youth	Program administrator Service providers Youth clients	1–3, 7,8,11,12	Relationship of YFHS with service integration
		Adapted from Philliber Research and Associates checklist for assessing teen friendliness of family planning services			
Dickson et al. (2007) [24]	South Africa; Upper middle income National Adolescent Friendly Clinic Initiative (NAFCI)/LoveLife clinics and control clinics	Quasi-experimental case-control study 1. Interviews with clinic manager 2. Clinic document review 3. Inventory of clinic 4. Provider interviews 5. Non clinical support staff interviews 6. Client-provider observations 7. Client exit interviews 8. Key informant interviews Adapted from NAFCI/LoveLife criteria	11 NAFCI clinics, 22 control clinics	1,2,5,7–12	Quality
Geary et al. (2014) [29]	South Africa; Upper middle income; 8 Primary health clinics	Cross-sectional study 1. Semi-structured interviews	8 nurses	1, 7, 10, 11	Perception
Geary et al. (2015) [25]	South Africa; Upper middle income; 15 health facilities	Cross-sectional study 1. Simulated clients debriefing questionnaires	50 visits by youth simulated clients	1–3,5,7,8,11,12	Perception and condom provision
Godia et al. (2014) [30]	Kenya; Low income; 9 facilities, youth center, district hospitals with integrative services	Cross-sectional study 1. Focus group discussions 2. In-depth interviews	18 focus group discussions 39 in-depth interviews of young people aged 10–24 years	1,2,5–11	Perception
Ingram and Salmon (2007) [26]	United Kingdom; High income; Drop-in primary care facility	Cross-sectional study 1. Questionnaires 2. interviews	122 young people aged 12–24 years	1, 5–8,11	Satisfaction
		Adapted from London School of Hygiene and Tropical Medicine Questionnaire for Service Users: Evaluation Kit			
Kavanaugh et al. (2013) [12]	USA; High income; Publically funded family planning facilities	Cross-sectional study 1. Close-ended questionnaires	584 Facility or agency directors	1,4,6,9,12	Contraception provision
		Adapted from Guttmacher family planning facilities tool			
Larke et al. (2010) [35]	Tanzania; Low income; 6 Health facilities	Simulated client clustered randomized control trial 1. Simulated client debriefing interviews	6 facility visits by youth mystery clients	4, 7,8,11	Quality and attendance, health seeking behavior, contraceptive distribution
Lesedi et al. (2011) [38]	Botswana; Upper middle income; 2 youth friendly clinics	Cross-sectional quantitative 1. Questionnaires	110 youth aged 15–29 years	1–3,5–12	Perception
		Adapted from Pathfinder Rapid Assessment of Youth Friendly Services (2003) 2. Mystery client interviews			
		Adapted from the African Youth Alliance Botswana interview guide (2005)			
Mashamba and Robson (2002) [27]	Zimbabwe; Low income; Youth advisory center	Cross-sectional study 1. Exit questionnaires 2. Focus group discussions	30 youth aged 15–24 years	1,2,4–10	Perception
Mathews et al. (2009) [31]	South Africa; Upper middle income; 12 NAFCI facilities and clinics	Cross-sectional study 1. Mystery client debrief questionnaires	137 youth mystery clients	6–8, 10	Compliance to quality criteria
		Adapted from UNAIDS (2002) and Family Health International VCT Toolkit			
Mauerhofer et al. (2010) [39]	Switzerland; High income; Multidisciplinary clinic	Cross-sectional study 1. Questionnaires	311 female return clients aged 12–20 years	1,2, 5–10	Satisfaction
		Adapted from WHO framework and Sovd et al. (2006) [34] study			
Mayeye et al. (2010) [40]	South Africa; Upper middle income; 11 Primary health clinics	Cross sectional study 1. Exit questionnaires	200 youth aged 16–19 years	1,2,4–11	Satisfaction and perception
		Adapted from International Planned Parenthood Federation Your Comments Count survey			
Mchome et al. (2015) [36]	Tanzania; Low income; 33 health facilities	Clustered randomized trial 1. Simulated client debriefings 2. interview checklist	48 visits by youth mystery clients	1,4,5,7,8,11	Quality
Mmari KN and Robert Magnani (2003) [33]	Zambia; Lower middle income; 10 health clinics	Quasi-experimental case control 1. In-depth interviews with managers, nurses, and staff 2. Focus group discussions with youth 3. Exit interviews with youth	200 youth in focus groups 60 youth interviews 10 Managers 20 Staff	1,3,4,7	Quality
		Adapted from the Pathfinder Focus on Young Adults Program			
Perry and Thurston (2007) [41]	United Kingdom; High income; 2 health facilities with youth only hours	Cross sectional study 1. Questionnaires	425 young people 10–18 years	1,2,4–8,10	Satisfaction
Sovd et al. (2006) [34]	Mongolia; Lower middle income; 51 YFHS facilities and 31 Controls	Quasi-experimental case control study 1. Exit survey questionnaires	1301 adolescents aged 10–19	1,2,5–10	Satisfaction and quality
		Adapted from collaboration with MOH, WHO and UNFPA			
Tanner et al. (2014) [32]	USA; High income; 15 clinics	Cross sectional study 1. Semi-structured interviews with staff 2. Photographs consistent and inconsistent with youth-friendliness	60 providers, outreach workers and case managers	3, 6–11	Perception
		Adapted from WHO Adolescent Friendly Health Services Agenda for Change Framework (2002)			

Legend:

^a^Setting described by study

^b^Domains:

1 = Accessible

2 = Acceptability

3 = Appropriate

4 = Equitable

5 = Effective

6 = Administrative procedure

7 = Staff characteristics and competency

8 = Confidentiality and Privacy

9 = Educational activities

10 = Environment

11 = Services provided

12 = Youth involvement

Of the 20 studies included in the review, 13 were located in sub-Saharan Africa, three in North America, three in Europe, and one in Asia. Six of the studies were conducted in high income countries and 14 in LMICs. Twelve of the studies were conducted with young people as respondents, three measured provider or staff responses, and five combined a mix of young people, provider, and non-clinical staff respondents.

No existing youth friendly assessment tool was used by more than one study, and no core set of indicators was commonly used between studies. Nine studies used a hybrid of questions from existing tools in addition to their own questions [23, 26, 31, 32, 34, 37–40]. Some YFHS assessments were based on those developed by international organizations such as the WHO, International Planned Parenthood Federation, UNAIDS, and Pathfinder International, as well as from national toolkits and institutes. Five of the six studies conducted in high income countries included in this review used distinct validated or existing tools in contrast to only half of the studies conducted in LMICs [12, 23, 26, 32, 39].

### Domains and indicators

A total of 115 indicators were identified in this review within 12 domains (Table 2). The three most frequently assessed domains were accessibility, staff characteristics and competency, and confidentiality and privacy. The most commonly used indicators are displayed in Table 3.

**Table 2:** Domains and Indicators for Assessing Youth-Friendly Services

Domains and Indicators	Total
Accessible^a^	18
• Convenient opening hours (*after school, weekends)*	14
• Distance/availability of transport to facility	11
• Services are affordable or free	10
• Outreach in the community	7
• Awareness of location, hours and services	5
• Appointment drop in available	5
• Dedicated services (*LARC insertion, HIV testing)* available at certain times of the day/week	3
• Youth-only hours	3
• Appointments available online or by text	2
• Social media presence for education and services	1
• Facilities open during entire posted time	1
• Partners welcome	1
Acceptable^a^	13
• General satisfaction	6
• Provider demographics reflect clients (*young, similar gender*)	6
• Client would recommend the clinic to friend	6
• Community members understand benefits and support provision of YFHS	3
• Client willingness to return to clinic	3
• Clinic has good reputation	1
• No corruption in facility	1
• All expectations of service are met	1
Appropriate^a^	8
• Package of care fulfills needs either at point of develiry or through referral linkages	7
• Client has choice of treatment options	2
• Data collected to determine young people’s health needs in community	1
Equitable^a^	8
• Welcome regardless of age	4
• Welcoming services for young men	3
• Open to all racial groups	1
• Open to all religious groups	1
• Welcome regardless of marital status	1
• Welcome regardless of relationship status	1
• Open to persons of all sexual orientations	1
• Females and males receive equal access to family planning services	1
• Males and females receive similar service care and respect	1
• Policies and guidelines for staff on SRH rights of young people	1
Effective^a^	8
• Supplies available onsite (*medical testing*)	6
• Providers are medically competent	2
• Provider takes client history	2
• Client follows caregivers advice, adherence to treatment	1
• Equipment to provide services available	1
• Process for ongoing quality improvement	1
• Client receives correct treatment	1
• Infection control procedures are followed	1
• Provider takes appropriate physical examination according to guidelines	1
Administrative procedures^b^	12
• Waiting times	9
• Choice and availability to be seen with same clinician during return visit	5
• Plan for follow up care explained and scheduled	4
• Referral care available, explained, and scheduled	4
• Sufficient time for consultation	3
• Frequency of appointments is convenient	1
• Do not need appointment for refills	1
• Number of times needed to return to clinic to obtain test results	1
• Hormonal contraceptive provision without appointment for pelvic exam	1
Staff characteristics and compentency	20
• Non judgemental	12
• Client recieves adequate information from provider	11
• Friendly	9
• Respectful	9
• Welcome/greeting	8
• Client has opportunity to ask all questions	7
• Listens to client problems	7
• Number of staff trained in YFHS	7
• Positive attitude	7
• Comfort in communicating	6
• Provider uses language that is understandable to clients	5
• Interested in client	3
• Willing to help	3
• Provider develops relationship with client	3
• Support and supervision for staff available on ongoing basis	3
• Responsive	2
• Client given time for test results to be absorbed and undertrstood	2
• Client is able to express opinion	2
• Provider answers questions to client’s satisfaction	2
• Explanation of services and treatment	1
• Training plan in place that meets needs of staff	1
• Provider perceives he/she has sufficient ability to provide services to youth	1
• Trustworthy	1
• Staff trained on how to communicate with teens over the phone	1
Confidentiality and Privacy	19
• Confidentiality is respected	8
• Client consultation cannot be heard or seen by other clients or staff	8
• Privacy is respected	6
• Staff explains services are confidential	4
• Parental consent is not required	3
• Consultation is not interrupted by outside staff or clients	3
• Passive disclosure of services avoided (*being seen in the waiting room discloses reason client is seeking service*)	3
• Tests are handled confidentialy	2
• Privacy asking for services in reception	1
• Staff uses shielded language when calling for appointment or follow-up	1
Educational Activities^b^	8
• Understandable and accurate SRH materials available	7
• Text message for follow-up or education	1
Environment^b^	12
• Comfortable	6
• Reading and/or entertainment materials available	4
• Clean	4
• Youth-only space	3
• Young people specific décor and materials	3
• Private waiting room for young people	2
• Ease of finding services within the facility	1
• Adequate lighting and ventilation	1
• Toilet facility quality	1
• Clean piped water	1
• Good phone access	1
• No overcrowding	1
Services Provided^b^	12
• Counselling (*prevention, condom demonstration, test results*)	7
• Contraceptive services	7
• STI services (*counselling, testing, treatment and prevention*)	7
• VCT available/HIV services	4
• Pregnant and parenting teen services	4
• Holistic approach (*services available beyond reproductive health including mental, psychosocial, lifeskills etc*)	3
• Pap smears and pregnancy tests	2
• Non-health services (*youth development services, domestic violence)*	2
• Emergency contraception	1
• Abortion services	1
• Mental heath services	1
• Treatment for minor ailments	1
Youth Involvement^b^	7
• Youth have input on service delivery	4
• Peer educator on staff	3
• Youth organize outreach	2
• Peer educator program in clinic	1

^a^Domains adapted from definitions provided by the World Health Organization: Quality Assessment Guidebook: A Guide to Assessing Health Services for Adolescent Clients [21, 19]

^b^Domains adapted from the definition provide by the Pathfinder Tool: Clinic Assessment of Youth-Friendly Services: A Tool for Assessing and Improving Reproductive Health Services for Youth [22, 20]

**Table 3:** The 10 most commonly used indicators for youth-friendly sexual and reproductive health services

1. Non-xjudgmental providers and staff	
2. Ease of access to location of facility	
3. Client receives adequate information from provider	
4. Services are affordable or free	
5. Staff is friendly	
6. Staff is respectful	
7. Reasonable waiting times	
8. Welcoming staff	
9. Confidentiality is respected	
10. Consultation cannot be heard or seen by other clients or staff	

Indicators were developed by researchers or identified by young people or staff respondents as measurements of youth-friendliness. We identified 44 unique indicators that were specific to only one study. The following section summarizes the indicators and frequency of inclusion within the twelve domains.

#### Accessibility

All but two of the studies assessed accessibility in some way [12, 23–30, 32–41]. This domain included three frequently used indicators: affordable or free services [12, 23, 26, 30, 34, 36–40], convenient opening hours [12, 23–30, 37–41], and ease of access to the facility [12, 23, 26–28, 30, 34, 37–39, 41]. Indicators for accessibility ranged from convenient location to young people’s awareness of services [12, 23–30, 32–41]. Only three studies, all of which were from high income countries, included youth-only hours as an indicator [23, 26, 38].

#### Acceptability

The included studies used eight different indicators for acceptability, with client’s willingness to recommend a clinic to a friend as the most common [23, 25, 34, 37–39]. Community acceptance of SRH services aimed at young people was also an important component of this domain [24, 29, 37]. Studies including only young people as participants typically assessed acceptance of the provider’s demographics, such as the provider being closer in age and having the same gender as the client [23, 25, 27, 28, 30, 40]. Measuring client satisfaction was commonly used to determine if health services were provided in a way that was perceived as acceptable by clients [25, 27, 28, 34, 39].

#### Appropriate

Only three indicators were used to measure the appropriate delivery of and inclusion of services to young people. The most common indicator for appropriate services was assessing the inclusion of youth-specific comprehensive care packages [23–25, 32, 33, 37, 38]. Studies that measured appropriateness described having policies and guidelines in place to fulfill the service needs of young people either at the point of delivery or through referrals and clinical linkages [23, 24, 29, 33, 35, 37, 38]. In addition, one study checked if the health needs of young people in the community had been assessed [24], and two assessed if clients were able to choose treatment options [24, 39].

#### Equitable

A common theme within this domain was gender equity, ensuring that males and females received the same standard and respect in the delivery of care [12, 25, 33, 36, 37]. In addition, some studies defined equitable services as those that did not discriminate based on relationship status [33, 37] or age [27, 33, 37]. One study assessed equity based on race, religion, and sexual orientation [41]. Studies assessed whether providers were judgmental towards young females wanting contraceptive services [25, 33, 36, 37] and whether services were welcoming to young men [25, 37]. Studies located in sub-Saharan Africa were more likely to include equity as a measurement than studies in other regions [27, 31, 33, 36, 37, 40].

#### Effective

To measure if health services were effective in providing care to young people, studies used indicators that related to following established protocols and factors that contributed to delivery of efficient health services. Only four studies measured the effectiveness of the service on client health outcomes such as contraceptive provision, condom use, and attendance [12, 25, 35, 37]. Effective providers were assessed by level of medical competency [30, 38], if they communicated in a way that the client could follow providers’ advice and treatment [39], if the client’s history was taken [24, 39], and if the correct treatment was received by the client [39]. Other indicators described facility characteristics that allowed for efficient delivery, including having supplies and equipment available on site [24, 30, 35, 36, 38, 40] and processes for quality improvement [24].

#### Administrative procedures

Distinct from the domain of accessibility, administrative procedures consisted of nine indicators relating to the choice to be seen with the same clinician during return visits [12, 28, 30, 39, 40] and scheduling of follow-up and referral care [22, 24, 29, 31, 32, 34, 36, 38, 40]. Long waiting times were a detriment to service delivery, and as such, was a frequent indicator in the domain [26, 27, 30, 31, 34, 37–39, 41].

#### Staff characteristics and competency

All studies included the characteristics of providers, receptionists, and/or other non-clinical staff as a domain for delivering youth-friendly services. Measurements of staff characteristics included interpersonal communication skills; an attitude that was friendly [26, 28, 30, 34, 37–41], positive [27, 28, 30, 31, 33, 36, 37], respectful [24, 25, 28, 31, 32, 35, 36, 38, 40], non-judgmental [24, 26, 28, 29, 31, 32, 35–40], and exhibiting comfort in communication [24–26, 28, 36, 39]. Other frequently used indicators include competency and the ability to deliver needed care to young people. Competency was measured through provider and client interactions such as listening to the client’s problems [25, 30, 31, 34, 36–40], answering questions [25, 41], providing adequate information [26, 28, 31, 33–35, 37, 38, 40, 41], and explaining procedures, treatment, and diagnosis [34]. Competency was also measured through initial staff training in youth-friendly services [12, 23, 24, 29, 35, 37, 40] and availability of opportunities for ongoing training [12, 24, 29].

#### Confidentiality and privacy

Nineteen of the 20 studies included an indicator for measuring confidentiality or privacy. Privacy tended to be measured in terms of a facility’s infrastructure. A common issue with consultation rooms and reception areas was the availability of a space that did not allow for other clients or staff to see or hear another client’s consultation and was not impeded by frequent interruptions during a consultation [12, 24, 25, 27, 30–32, 34–36, 38, 39, 41]. Confidentiality included confidential handling of tests [31] and avoiding passive disclosure of services [27, 32, 41]. Passive disclosure was the provision of services in a manner that allowed anyone who sees a client at the clinic to identify the reasons they were accessing services [32].

#### Educational activities

Educational activities were the second least commonly measured domain. Eight studies investigated educational activities as an aspect of YFHS using two indicators, availability of educational materials at the facility and the use of text messages for follow-up education. Materials that bolster SRH information received from the provider included informational pamphlets and other educational materials such as videos available in the clinic [24, 27, 30, 32, 39, 40]. One study measured the use of text messages as a follow-up educational tool [21].

#### Environment

The environment domain was primarily described as a clinic that feels comfortable, with indicators that varied by study and setting [23, 24, 27, 30–32, 34, 38–41] Youth specific décor [12, 32, 38], cleanliness [24, 30, 34, 40], youth-only spaces [29, 30, 32], and reading and entertainment material [30, 32, 34, 40] were used to describe comfort in youth-friendly clinics. Eight of the twelve indicators were not specifically related to a youth-only environment such as such as lighting, ventilation, and toilet facility quality [24, 27, 29–31, 34, 38–41].

#### Services provided

Twelve indicators were used to assess services provided at youth-friendly clinics. The most common measurements included SRH counselling on topics including contraceptive education, condom demonstrations, and test results [23, 24, 29–31, 35, 36, 38–40]. One paper analyzed the relationship between youth-friendliness and integration with other primary care services [23] and five studies assessed if the facility integrated other services with SRH. [23, 26, 30, 38, 40].

#### Youth involvement

The participation of young people within YFHS was the least used domain in terms of measuring YFHS and was included in seven studies located in upper middle income and high income countries [12, 23, 24, 29, 32, 37, 38]. Youth involvement pertained to young people as educators on staff [12, 25, 37], outreach organized by young people [23, 37], and young people having the opportunity to be part of the service delivery design and evaluation [23, 24, 37, 38].

## Discussion

This systematic literature review identified indicators that have been used to define and measure youth-friendly SRH services from the perspectives of researchers, young people providers, and non-clinical staff. From this review, we identified 12 domains that encompassed 115 YFHS indicators. Three domains stood out as frequently used to assess the delivery of SRH services to young people: *accessibility of services, privacy and confidentiality, and staff characteristics and competencies*. While the broadness of the WHO framework of “accessibility, acceptability, appropriateness, equitability, and effectiveness” would likely incorporate most indicators, our study further expands on those domains. Our findings elaborate on previous literature results and underline how youth-friendly SRH services are being measured in a range of settings and from different perspectives of care. Although it is beyond the scope of this review to determine the most appropriate measures for YFHS, our review found a series of indicators that may have little relevance for youth-specific or SRH-specific measurements such as clean water or ventilation. These may be important indicators to measure overall quality or basic standards of care for all ages and a variety of health needs, but they also highlight a need to prioritize indicators based on greatest importance to young people.

The studies included in the review did not use similar tools or indicators to assess YFHS. In fact, almost two-fifths of the indicators were unique to the specific study. While there are many tools available for assessing YFHS, no study used the same tool. This variation highlights the need to standardize the way in which YFHS are measured with a minimum of a core set of indicators to enable findings to be more easily compared across settings. This variability has limited the comparability and generalizability of assessments of the effectiveness of youth-friendly services. While standardized tools will provide a core set of indicators, these tools may need to be adapted or augmented for different cultural contexts [42, 43]. The presence of unique indicators may suggest that there are distinct contextual settings for health service delivery or may reflect a researcher’s particular interests. For example, indicators in the domain of confidentiality and privacy and the domain of staff characteristics and competency were included in all studies in this review, but measured differently. Indicators in other domains, such as young people’s involvement and educational activities, may need further development, adoption, and more widespread use if prioritized as important for YFHS. In each case, researchers and practitioners need to reach a consensus on the priorities and the specific measurements. Given the importance of their perspective, young people should also play a role in providing input in the development of priorities and measures. In addition to providers and youth input, community stakeholders, researches, NGOs, and public institutions can play a collaborative role in establishing these priorities [42, 44, 45].

We expected that youth-specific indicators would have featured more prominently in our results for measuring youth-friendly SRH services. Young people’s involvement in YFHS development, delivery, and evaluation, along with appropriate environments, were the least likely indicators to be measured. The ten most commonly used indicators to measure YFHS in the identified studies (Table 3) emphasized convenient opening hours, nonjudgmental attitudes, ease of access to location of facility, receipt of adequate information from providers, services that are affordable or free, friendly and respectful staff, reasonable waiting times, and confidentiality. It is notable that while some indicators such as confidentiality and nonjudgmental attitudes are important in the delivery of SRH services, none of these indicators is specific to youth-friendliness and may be more relatable to general access and quality of care, an underlying theme in the assessment of indicators in this review. Improvements in providing health care overall can coincide with increased services utilization among adolescents [46]. Similarly, many of the indicators related to clinic environment were not specific to youth-friendliness and were more a measure of basic facility standards (e.g., clean water) or services (e.g., not corrupt).

Some evidence also suggests that young people may not prioritize what providers and programmers view as youth-specific approaches [47], such as youth-only spaces, entertainment, and welcoming décor; they may prioritize issues such as confidentiality and costs [16] far more. For example, a survey of youth preferences in Kenya and Zimbabwe found young people valued integrated services, low-cost service, short wait times, and staff with friendly attitudes [16]. In contrast, young people did not prioritize choosing services that assured youth-specific spaces such as youth-only and single-sex only facilities [16]. Some studies have found that youth-only spaces may not be effective in increasing service use [16] as young people may realistically fear the stigma associated with seeking SRH care, given negative views and societal values about sexuality of young people [48]. Lack of clear prioritization among measurements of youth-specific SRH needs suggests the term “youth-friendly” may often be no more than an attempt by clinical and public health professionals to develop what they perceive to be attractive services. In part, the absence of a standardized tool or indicators for measuring youth-friendly SRH services is indicative of a vague definition of exactly what constitutes YFHS, but also why there is limited evidence for the effectiveness of YFHS.

The results show that little is known about precisely what dimensions are most needed to serve the SRH needs for young clients and whether the most relevant indicators would vary from the indicators used to measure services for adults or younger children, or services for primary care versus SRH services. For example, issues of confidentiality may be more important to young people given cultural attitudes regarding pre-marital sexual relationships. Furthermore, no studies examined the use of different services to assess uptake or to differentiate age-specific needs such as education or long acting contraceptive counselling. Only one study mentioned LGBTQ youth, suggesting future research needs to understand if there are specific service needs for LGBTQ youth and other marginalized sub-populations and to what extent these needs are being met [47]. Understanding which domains are most important in delivering health services to young people when there are only finite resources available needs to be further assessed.

Our review also confirmed that evidence of the impact of YFHS on SRH service use and health outcomes is limited. Other reviews assessing the effectiveness of research on YFHS similarly found the need for a more rigorous approach to developing and using tools to test the effectiveness of YFHS strategies on health outcomes [15, 18].

### Limitations

Our review found only a small number of studies that met the inclusion and exclusion criteria, which may limit the generalizability of this review. No studies from Latin America and only one study from Asia were included in our review. These results could be due to the language restrictions to English publications used in this review. However, previous systematic reviews on YFHS also did not yield any results in Latin America [15, 18]. Because these regions were not included in the review, the relevance of these indicators in LMICs not located in sub-Saharan Africa is not known. We did not include grey literature in our review where additional multilateral and non-governmental organization evaluations of YFHS may be published. We also chose to include studies of limited rigor because they were still relevant to assess how youth-friendly services were being defined and measured even though they provide weak evidence of effectiveness. A quality assessment of the included studies was not conducted for this review because the aim was to gather and analyze the type of measurements used for evaluating YFHS and not to focus on the outcomes.

### Conclusion

This review identified the range of indicators and domains used to measure youth-friendly SRH services. The set of indicators collected in this review can provide a framework for how to further define, standardize, and evaluate the core components of SRH services for young people. These indicators, while comprehensive, require further refinement and further development to determine and compare the effectiveness of YFHS initiatives globally. Future research needs to use a set of core indicators in addition to location- and culturally specific measures to assess youth-friendliness and determine which specific aspects improve health service delivery, service utilization, and health outcomes. This research could inform administrators, managers and policymakers where to allocate resources most efficiently. Worldwide, governments are adapting national standards for YFHS, however, concrete evidence supporting such policy shifts and allocation of resources is needed.

## Abbreviations

LMICs: Low and middle income countries
NAFCI: National Adolescent Friendly Clinic Initiative
RCTs: Randomized control trials
SRH: Sexual and reproductive health
STIs: Sexually transmitted infections
VTC: Voluntary testing and counseling
WHO: World Health Organization
YFHS: Youth-friendly health services
